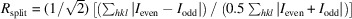# A simple goniometer-compatible flow cell for serial synchrotron X-ray crystallography

**DOI:** 10.1107/S1600576723001036

**Published:** 2023-03-09

**Authors:** Swagatha Ghosh, Doris Zorić, Peter Dahl, Monika Bjelčić, Jonatan Johannesson, Emil Sandelin, Per Borjesson, Alexander Björling, Analia Banacore, Petra Edlund, Oskar Aurelius, Mirko Milas, Jie Nan, Anastasya Shilova, Ana Gonzalez, Uwe Mueller, Gisela Brändén, Richard Neutze

**Affiliations:** a Department of Chemistry and Molecular Biology, University of Gothenburg, Medicinaregatan 9C, 40530 Gothenburg, Sweden; b MAX IV Laboratory, Lund University, Fotongatan 2, 224 84 Lund, Sweden; c Diamond Light Source, Harwell Science and Innovation Campus, Didcot OX11 0DE, United Kingdom; d Macromolecular Crystallography Group, Helmholtz-Zentrum Berlin, Albert-Einstein-Strasse 15, 12489 Berlin, Germany; Universität Hamburg, Germany

**Keywords:** serial synchrotron X-ray crystallography, macromolecular crystallography, cytochrome *c* oxidase, goniometer-compatible flow cells

## Abstract

Serial femtosecond crystallography was initially developed for room-temperature X-ray diffraction studies of macromolecules at X-ray free electron lasers. When combined with tools that initiate biological reactions within microcrystals, time-resolved serial crystallography allows the study of structural changes that occur during an enzyme catalytic reaction. Serial synchrotron X-ray crystallography (SSX), which extends serial crystallography methods to synchrotron radiation sources, is expanding the scientific community using serial diffraction methods. This report presents a simple flow cell that can be used to deliver microcrystals across an X-ray beam during SSX studies. This device consists of an X-ray transparent glass capillary mounted on a goniometer-compatible 3D-printed support and is connected to a syringe pump via light­weight tubing. This flow cell is easily mounted and aligned, and it is disposable so can be rapidly replaced when blocked. This system was demonstrated by collecting SSX data at MAX IV Laboratory from microcrystals of the integral membrane protein cytochrome *c* oxidase from *Thermus thermophilus*, from which an X-ray structure was determined to 2.12 Å resolution. This simple SSX platform may help to lower entry barriers for non-expert users of SSX.

## Abstract 2

Serial crystallography, in which X-ray diffraction data are recorded from a sequence of microcrystals in serial fashion, requires a platform for sample delivery. This work describes a simple flow cell device designed to deliver protein microcrystals to an X-ray beam during serial X-ray crystallography studies using synchrotron radiation.

## 1. Introduction

Serial crystallography describes an experimental approach in which a sequence of X-ray diffraction images is collected while continuously delivering new randomly oriented microcrystals to the X-ray beam at synchrotron sources or X-ray free-electron lasers (XFELs) (Chapman *et al.*, 2011 ▸; Boutet *et al.*, 2012 ▸; Schlichting, 2015 ▸). When using XFEL radiation (Chapman *et al.*, 2011 ▸), it was proposed that the X-rays would diffract from the sample before X-ray induced radiation damage destroyed the ability of the sample to diffract (Neutze *et al.*, 2000 ▸). This concept became known as ‘diffraction before destruction’ (Chapman *et al.*, 2014 ▸) and underpins the need for sample injection technologies that continuously replace the sample (Weierstall *et al.*, 2012 ▸). The serial approach was later extended to synchrotron sources (Gati *et al.*, 2014 ▸; Nogly *et al.*, 2015 ▸; Meents *et al.*, 2017 ▸; Stellato *et al.*, 2014 ▸) and is termed serial synchrotron X-ray crystallography (SSX) (Diederichs & Wang, 2017 ▸).

In contrast with widely applied methods of cryocrystallography, in which X-ray diffraction data are recorded from a single crystal cooled to cryogenic temperature and rotated during data collection, serial crystallography studies are almost always performed at room temperature. This allows biological reactions to be initiated in microcrystals, and time-resolved diffraction can then be used to study structural changes during enzyme catalysis (Branden & Neutze, 2021 ▸). Indeed, time-resolved SSX (Pearson & Mehrabi, 2020 ▸; Weinert *et al.*, 2019 ▸; Mehrabi *et al.*, 2019*b* ▸) has been developed to complement time-resolved diffraction studies using XFEL radiation (Tenboer *et al.*, 2014 ▸). In serial crystallography, all diffraction images are derived from randomly oriented samples, and it is usually necessary to collect thousands of diffraction images in order to recover high-quality crystallographic data sets. When using a polychromatic rather than a monochromatic X-ray beam it is possible to significantly reduce the required number of X-ray diffraction images (Meents *et al.*, 2017 ▸). Data collected from microcrystals are indexed, integrated and merged using software developed for this purpose (White *et al.*, 2012 ▸).

With continuous improvements in the performance of storage-ring X-ray sources (Castelvecchi, 2015 ▸), high-fluence micro-focused X-ray diffraction instruments suitable for SSX studies are becoming widespread. Since there are many more synchrotron radiation facilities than XFELs, and each synchrotron source often has several macromolecular crystallography beamlines supporting external user experiments, there exists a large capacity for growth in the use of SSX. However, there remain significant entry barriers for inexperienced users, such as the need to grow large volumes of micrometre-sized crystals and to learn new data-processing software. Mastering sample delivery technologies also places a significant overhead on new users of SSX, and consequently a small number of experienced users have tended to dominate the SSX literature to date (Oghbaey *et al.*, 2016 ▸; Beyerlein *et al.*, 2017 ▸; Mehrabi *et al.*, 2020 ▸; Monteiro *et al.*, 2020 ▸; Nogly *et al.*, 2015 ▸; Schulz *et al.*, 2018 ▸; Sherrell *et al.*, 2015 ▸; Weinert *et al.*, 2017 ▸; Botha *et al.*, 2015 ▸). Thus, although many sample injection technologies are mature, they have not yet fully engaged the broader macromolecular crystallography community.

Sample delivery technologies for serial crystallography studies broadly fall into two classes: fixed-target platforms, where microcrystals are pre-mounted on a platform and are continuously replaced by translating the target mount (Sherrell *et al.*, 2015 ▸; Mueller *et al.*, 2015 ▸; Mehrabi *et al.*, 2019*a* ▸, 2020 ▸; Schulz *et al.*, 2018 ▸; Suga *et al.*, 2019 ▸), and flowing systems, including liquid and high-viscosity microjets (Weierstall *et al.*, 2014 ▸, 2012 ▸; DePonte *et al.*, 2008 ▸; Vakili *et al.*, 2022 ▸, Weinert *et al.*, 2017 ▸) or microfluidic devices (Monteiro *et al.*, 2020 ▸), in which the sample is continuously replaced as it flows through a fixed injector. Tape conveyor belts have also been developed which combine some benefits of fixed-target and flowing technologies, whereby microcrystal droplets are dispensed onto a continuously moving tape which translates these droplets through the X-ray beam (Fuller *et al.*, 2017 ▸; Beyerlein *et al.*, 2017 ▸). All systems have their specific advantages and disadvantages. Fixed-target systems use very little sample but must be regularly replaced, and this usually requires entering the hutch. In some cases, microcrystals have been grown directly on fixed-target platforms (Opara *et al.*, 2017 ▸; Lieske *et al.*, 2019 ▸; Broecker *et al.*, 2018 ▸; Norton-Baker *et al.*, 2021 ▸; Murray *et al.*, 2015 ▸). Flowing systems may be operated continuously for longer periods of time. Microjet technologies have also been widely used in time-resolved diffraction studies (Branden & Neutze, 2021 ▸) but can be demanding in terms of sample consumption, although this problem can be alleviated when it is possible to use viscous carrier media and thereby flow the sample at a slower rate (Nam, 2022 ▸; Weierstall *et al.*, 2014 ▸; Sugahara *et al.*, 2015 ▸). Many synchrotron radiation sources and all XFEL sources offer sample delivery support for serial crystallography to new and experienced users. However, for complex experiments including time-resolved X-ray diffraction studies, preparative work with the sample delivery system may be essential and requires either additional travel to the X-ray facility or a large upfront investment in the necessary infrastructure for in-house studies.

When using synchrotron radiation, a technically simpler method than microjets for sample delivery is to use an X-ray transparent glass capillary and collect X-ray diffraction data as microcrystals flow through the enclosed sample delivery environment. The flow cell systems reported to date (Stellato *et al.*, 2014 ▸; Nam, 2020 ▸) required mounting and alignment stages that are independent of the standard macromolecular crystallography endstation configuration and therefore involved an additional overhead of building and dismounting the sample stage. Moreover, the sample stage design must accommodate the spatial constraints of the endstation and may not always be easy to move between different macromolecular crystallography beamlines. With these limitations in mind, we designed a flow cell that is mounted on a standard magnetic mount, such as the European SPINE, as used at synchrotron-based macromolecular crystallography beamlines. This device consists of an X-ray transparent glass capillary which is mounted on a lightweight 3D-printed base supporting an iron or magnetized disc held by friction. Flexible and lightweight fused silica capillary tubing is used to deliver slurries of microcrystals from a syringe, through the mounted X-ray transparent capillary and across the X-ray beam, before the sample is collected in a 3D-printed catcher. This design exploits the alignment tools of the macromolecular beamline without the need for any additional construction or sample-alignment hardware, and little time is lost when exchanging samples or if the system becomes blocked. The device was successfully demonstrated in SSX studies using microcrystals of the integral membrane protein cytochrome *c* oxidase (C*c*O) from *Thermus thermophilus.* Serial crystallographic data yielded an SSX structure of C*c*O to 2.12 Å resolution, which compares favourably with an earlier SFX structure determined using XFEL radiation (SACLA, Japan) (Andersson *et al.*, 2017 ▸). We suggest that this convenient, lightweight and replaceable system for sample delivery can help to lower entry barriers for researchers without previous experience in serial crystallography and provides a framework for routine collection of serial crystallography data using synchrotron radiation.

## 2. Methods

### 2.1. Design of a 3D-printed plug for flow cell assembly

**Figure 1:**
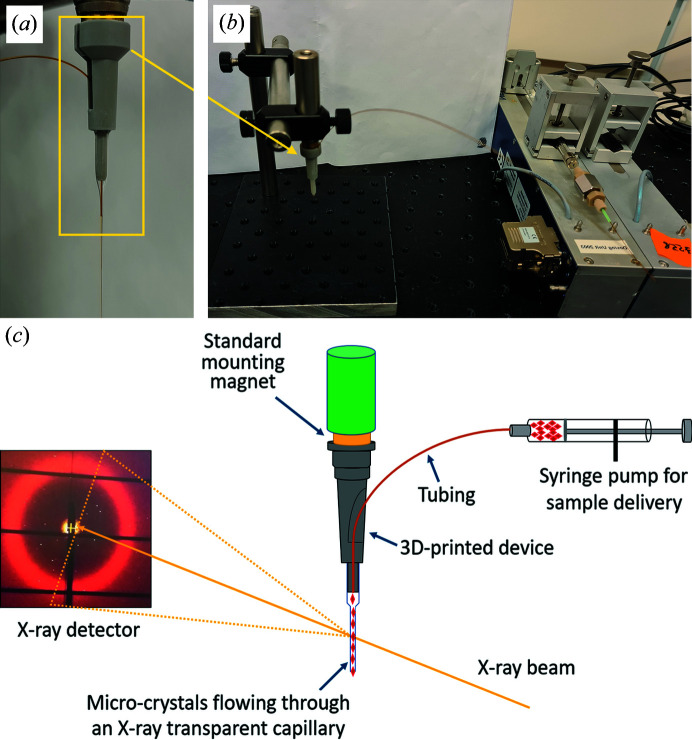
Overall design of the 3D-printed flow cell. (*a*) Flow cell with a 3D-printed base glued to an X-ray transparent glass (or quartz) capillary containing a channel through which a fused silica capillary tube is inserted and through which the sample is transported. This device is supported by a magnetic connection (either an iron or magnetized bullet) held by friction. The dimensions of the base can be adjusted to be compatible with any goniometer magnet used at any synchrotron radiation source. (*b*) Flow cell device connected to a syringe pump using suitable connectors. (*c*) Schematic of how the flow cell is used as a sample delivery system at a macromolecular crystallography beamline. Microcrystals (red) are injected from a syringe connected to a pump and delivered into an X-ray beam through an X-ray transparent capillary held in place on a standard goniometer magnet. X-ray diffraction data from microcrystals are collected on a rapid-readout X-ray detector.

A lightweight base for the flow cell was designed to fit within the space limitations of a standard goniometer sample mounting system using the *AutoCAD* (Autodesk 2020) software. Prototypes were printed on a 3D printer (MAX/MAX UV, Asiga technologies) using plastic resin (Asiga PlasGRAY V2) [Figs. 1 ▸(*a*) and 1 ▸(*b*)] and the design was iteratively improved. After printing, each base was washed with iso­propanol and the internal channels were purged with iso­propanol-filled syringes to remove any residual resin. The system was then dried in air and subjected to two steps of hardening using UV light (2 × 2000 flashes).

**Figure 2:**
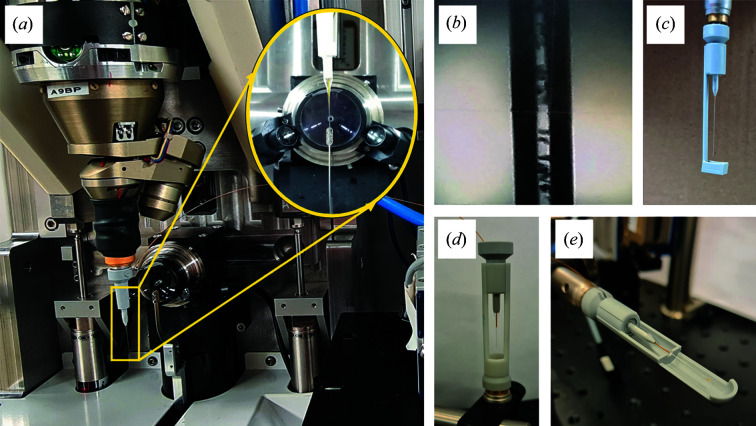
Mounting and alignment of the 3D-printed flow cell on a macromolecular crystallography beamline. (*a*) Flow cell mounted on the goniometer magnet and aligned with the X-ray beam at BioMAX. (*b*) LCP crystals of *ba*_3_-type C*c*O injected into the flow cell were observed through the glass capillary using the standard alignment optics of BioMAX. (*c*) Design of the catcher when the goniometer allows vertical mounting from above. This catcher is mounted by sliding over the flow cell and is held in place by friction. (*d*) Design of the catcher when the goniometer allows vertical mounting from below. In this case the sample also flows downwards. (*e*) Design of the catcher when the goniometer allows horizontal mounting.

Each 3D-printed component contains a base that can grip a magnetic disc (5 × 5 mm, Supermagnet, Neodymium N45, S-05-05-N), or a similar-sized iron disc, allowing the system to be mounted on a goniometer magnet and aligned to the X-ray beam using the alignment tools of the beamline. This base is assembled to form a flow cell device by first inserting fused silica capillary tubing (TSP250350, CM scientific Polymicro technologies) through a pore within the 3D-printed base and then glueing them using superglue. This tubing typically has an outer diameter of 360 ± 10 µm and an inner diameter of 250 ± 6 µm and is cut at a length that allows it to reach from the syringe pump to the magnetic mount [Figs. 1 ▸(*b*) and 1 ▸(*c*)]. This geometry requires that the fused silica capillary tubing enters from the side of the 3D-printed base. As such, an important consideration of this design was that the curvature of any pore within the 3D-printed base was sufficiently low that the fused silica capillary tubing did not break, while being sufficiently high to enable the flow cell to be mounted on a standard goniometer magnet and maintain a standard operational distance of 25 to 30 mm from the magnetic base to the X-ray beam. The assembly of the flow cell is completed by glueing a thin X-ray transparent borosilicate glass capillary (Hampton Research Glass Number 50 Capillary) onto the opposite end of the 3D-printed device to the magnet [Figs. 1 ▸(*a*) and 1 ▸(*c*)]. In this work glass capillaries of 100 and 200 µm in diameter are evaluated, although other operational diameters for the glass capillary may be chosen if desired. The wall thickness of these glass capillaries is nominally 10 µm, and the glass capillary is typically cut at a distance of 50 to 55 mm from the magnetic cap. An assembled flow cell weighs less than 3 g, and pre­assembled flow cells can be transported to the synchrotron radiation facility using 3D-fabricated supports. During X-ray diffraction data collection [Figs. 2 ▸(*a*) and 2 ▸(*b*)], a 3D-printed catcher is slid over the flow cell around the glass capillary to maintain a clean work environment, and this collects sample which has been exposed to the X-ray beam as it emerges from the cut glass capillary [Figs. 2 ▸(*c*) and 2 ▸(*e*)].

The assembled and mounted flow cell is connected via fused silica capillary tubing to a gas-tight Hamilton syringe using compatible sleeves, connectors and unions to avoid sample leakage. We employed commercially available tight-fitting sleeves (∼1 cm, IDEX F-242X) and connectors (IDEX F-120x and F-333Nx+F142Nx) that fit into a union (IDEX P-742) in order to connect to a 100 µl Hamilton syringe needle. The syringe is connected to a syringe pump (CETONI Low Pressure Syringe Pump Nemesys 290 N) and delivers sample to the capillary using a program available with the operating software (Nemesys *UserInterface* software) [Fig. 1 ▸(*b*)]. During SSX data collection, the syringe pump was operated from the control room of the synchrotron beamline.

### 2.2. Sample preparation for SSX and spectroscopy

Proof-of-principle experiments were conducted at the MAX IV Laboratory (Lund, Sweden) by collecting SSX data from a sequence of microcrystals of *ba*_3_-type C*c*O from *T. thermophilus*. The protein was produced, purified and crystallized via methods described previously (Andersson *et al.*, 2017 ▸) using a well founded technique for large-scale production of *ba*_3_-type C*c*O microcrystals (Andersson *et al.*, 2019 ▸). Purified protein was concentrated to 12 to 15 mg ml^−1^ in 20 m*M* Tris–HCl pH 7.6, 0.05%(*w*/*v*) DDM, 80 m*M* NaCl. The protein concentration was determined by reducing the enzyme with excess sodium di­thio­nite, measuring the absorbance at 560 and 590 nm, and using the relationship that the absorbance difference Δɛ_560−590_ = 26 000 *M* ^−1^ cm^−1^ (Chen *et al.*, 2005 ▸). For the lipidic cubic phase (LCP) crystallization, concentrated protein was mixed with monoolein [9.9 mono­acyl­glycerol (MAG), Nu-Check Prep; CAS 111-03-5] at a ratio of 2:3 protein to lipid (40 µl of protein and 60 µl of monoolein) using an LCP coupler connected to two gas-tight 100 µl syringes (Hamilton, Model 1710 RN SYR, small removable needle, 22 gauge) (Caffrey & Porter, 2010 ▸). Strings of LCP reconstituted protein (10 to 15 µl) were then dispensed into a nine-well glass plate containing 300 µl of 1.4 *M* NaCl, 100 m*M* MES pH 5.3 with 36 to 39%(*v*/*v*) PEG 400. The plate was covered with a transparent seal (Molecular Dimensions, ClearVue TM Sheets, MD6-O1S, Lot No: 11024) and micro-crystals of 5 to 30 µm in their longest dimension were obtained at room temperature within 2 to 3 days of incubation in the precipitant solution. Crystallization batches containing microcrystals from 15 to 25 µm in dimension were chosen for SSX studies. LCP crystals of *ba*_3_-type C*c*O were transported in 500 µl syringes (Hamilton, 500 µl Gastight Syringe Model 1750 RN, large removable needle, 22 gauge) to the MAX IV Laboratory for SSX data collection. The LCP phase was softened by the addition of 5 to 10 µl of PEG 400 into the syringe immediately prior to sample injection.

Since the flow cell design is airtight, it was also possible to prepare the enzyme in different reduction states for spectroscopic characterization, using samples both in solution and in microcrystalline form. Steady-state absorption spectra of continuously flowing samples of oxidized and reduced forms of *ba*_3_-type C*c*O in solution and *in crystallo* were measured and analysed using a microspectrophotometer modified from an earlier design (Hadfield & Hajdu, 1993 ▸) with the flow cell held in position using a magnetic mount. Reduced samples of C*c*O were purged with N_2_ gas and treated with sodium di­thio­nite before loading into a 100 µl Hamilton syringe. The setup was incubated with sodium di­thio­nite for 15 to 20 min and washed with de­oxy­genated buffer to achieve an oxygen-free condition prior to the delivery of reduced C*c*O samples.

### 2.3. SSX data collection and structure determination

SSX data were collected using the 3D-printed flow cell at the BioMAX beamline of the MAX IV Laboratory (Ursby *et al.*, 2020 ▸). A preassembled flow cell was mounted on the goniometer magnet and aligned with the X-ray beam using the in-line visualization tools of the beamline [Figs. 2 ▸(*a*) and 2 ▸(*b*)], and this step took only a few minutes in total. A 100 µl Hamilton syringe was loaded with LCP crystals of *ba*_3_-type C*c*O and installed on a CETONI Nemesys syringe pump. Sample viewing, alignment and measurement were carried out using the beamline control software *MXCuBE3* (Mueller *et al.*, 2017 ▸). Data collection was triggered manually when sample was observed to flow through the glass capillary [Fig. 2 ▸(*b*)]. After exposure to the X-ray beam, samples were collected in a catcher [Figs. 2 ▸(*c*)–2 ▸(*e*)].

X-ray diffraction data were collected at room temperature using a full width at half-maximum X-ray beam size of 5 µm (V) × 20 µm (H), a photon energy of 12.6 keV and a flux of 3.6 × 10^12^ photons s^−1^. LCP crystals of *ba*_3_-type C*c*O were injected vertically downwards [Fig. 2 ▸(*a*)] at flow-rates of 0.3 µl min^−1^ for the 100 µm-diameter glass capillary and 1.2 µl min^−1^ for the 200 µm-diameter glass capillary. In both cases this equates to a downward velocity approximately equal to 0.64 mm s^−1^, and it therefore took approximately 8 ms for the sample to move through the X-ray beam. Since it is this transit time through the X-ray beam that determines the X-ray exposure per crystal, we calculate an average radiation dose for each crystal of 40 kGy using *RADDOSE-3D* (Bury *et al.*, 2018 ▸; Paithankar *et al.*, 2009 ▸). X-ray diffraction data were recorded on an EIGER 16M hybrid pixel detector at a frame rate of 20 Hz, or 50 ms per frame. Interaction with the X-ray beam led to the charring of the glass capillary over time, with a small amount of protein precipitation accumulating on the inner walls of the capillary. This issue was periodically addressed during data collection by translating the capillary approximately 100 µm in the vertical direction using the standard alignment tools of the beamline.

Diffraction data were indexed, integrated, merged and converted to MTZ format using *CrystFEL* (version 0.10; White *et al.*, 2012 ▸, 2016 ▸). Indexing rates of 13.9 and 48.6% were recovered for data collected using 100 and 200 µm-diameter X-ray capillaries, respectively. Data truncation, phasing and structural refinement were performed using the *CCP4i* suite (Winn *et al.*, 2011 ▸) in the *CCP4 Cloud* (Krissinel *et al.*, 2022 ▸). An SFX structure of *ba*_3_-type C*c*O (PDB entry 5ndc; Andersson *et al.*, 2017 ▸) was used as a model for mol­ecular replacement with *Phaser* (McCoy *et al.*, 2007 ▸). Data collected from the 100 µm-diameter glass capillary were cut at 3.05 Å resolution and were not used for structural refinement. The SSX structure of *ba*_3_-type C*c*O in LCP was refined to 2.12 Å from data collected using the 200 µm-diameter glass capillary employing one round of rigid body refinement in *REFMAC5* (Murshudov *et al.*, 2011 ▸) followed by several rounds of restrained refinement including TLS refinement (Winn *et al.*, 2001 ▸). Model building was performed in *Coot* (Emsley & Cowtan, 2004 ▸). Composite omit maps were calculated in *PHENIX* (Liebschner *et al.*, 2019 ▸). All structural representations were drawn in *PyMOL* (version 1.2r3pre, Schrödinger, LLC; http://www.pymol.org). Data collection and refinement statistics are given in Table 1 ▸.

## 3. Results and discussion

### 3.1. Rapid mounting and alignment of the flow cell for SSX

Our magnetically mounted flow cell was designed to respect the rather stringent space limitations surrounding a macromolecular crystallography goniometer. This was achieved by requiring that samples are transported through fused silica capillaries (inner diameter of 250 µm) from a syringe pump conveniently placed relative to the mounted capillary. This use of transport lines creates the potential for pressure accumulated along these lines leading to sample blockage. Validation studies using viscous LCP crystallization media show that viscous samples can be transported over lines up to 50 cm in length, although longer transport lines lead to a larger dead volume (∼0.5 µl cm^−1^). Since the system is entirely enclosed, the glass-capillary walls support the flowing medium, preventing it from curling up or aggregating when exposed to X-rays, and there is no need for a focusing outer gas stream, as is required for liquid (DePonte *et al.*, 2008 ▸; Weierstall *et al.*, 2012 ▸) or high-viscosity (Weierstall *et al.*, 2014 ▸; Sugahara *et al.*, 2015 ▸) injectors. There are therefore few negative consequences when using additives to reduce viscosity and thereby improve the flow of the sample. Indeed, the flow cell can support entirely liquid microcrystalline slurries delivered at flow-rates up to two orders of magnitude lower than used for liquid gas dynamic virtual nozzle injectors (DePonte *et al.*, 2008 ▸; Weierstall *et al.*, 2012 ▸). However, as with liquid-jet and some fixed-target systems, microcrystal settling can become a problem if the transport medium cannot support the microcrystals in suspension for the full duration of data collection.

It typically took a few minutes to connect the fused silica transport capillaries to a syringe, mount this within a syringe pump, mount the flow cell on the goniometer and align it. Since the sample volume per injection is set by the choice of syringe and the flow-rate may be low (here we used 0.3–1.2 µl min^−1^), it was possible to collect SSX data uninter­rupted for more than an hour at a time. Blockages occurred more frequently when using the 100 µm-inner-diameter glass capillaries than the 200 µm capillaries. Although the pressures reached within our system may be expected to be less than those used in a high-viscosity injector (Weierstall *et al.*, 2014 ▸; Shimazu *et al.*, 2019 ▸), which is usually operated with a 75 or 50 µm nozzle, the Hamilton syringe and associated connections are a weak point where leaks may arise under pressure, and on rare occasions the 3D-printed device could also crack. Moreover, since the system is enclosed, the X-ray beam can cause aggregated protein to accumulate on the inside of the capillary and, over time, this may increase the risk of a blockage. Nevertheless, our experience is that between one and four flow cell devices typically served a 24 h beam time allocation, depending on the propensity of the sample to cause blockages. These properties of the goniometer-mounted flow cell combine to allow any macromolecular crystallography beamline with a sufficiently rapid X-ray detector readout to be used efficiently for SSX data collection.

### 3.2. High-resolution room-temperature SSX structure of *ba*_3_-type C*c*O

Proof-of-principle demonstrations of the practical value of the flow cell for SSX data collection were performed using microcrystals of the integral membrane protein cytochrome *c* oxidase from *T. thermophilus*. This enzyme belongs to a large family of terminal oxidases, which accept electrons to reduce molecular oxygen to water during cellular respiration. Considerable biochemical and biophysical data, including a number of resting state structures of *T. thermophilus* C*c*O, have yielded insight into the function of this enzyme (Soulimane *et al.*, 2000 ▸; Tiefenbrunn *et al.*, 2011 ▸; Chang *et al.*, 2009 ▸; von Ballmoos *et al.*, 2015 ▸; Siletsky *et al.*, 2007 ▸), although structural details concerning the mechanism of redox-linked proton translocation remain elusive. Serial crystallography studies have previously reported an SFX structure of *T. thermophilus* C*c*O to 2.3 Å resolution (PDB entry 5ndc) using a 75 µm-diameter nozzle in a high-viscosity microjet injector at SACLA (Andersson *et al.*, 2017 ▸), and an SSX structure was recovered at 3.6 Å at MAX IV Laboratory using the high-viscosity extrusion injector with a 100 µm-diameter nozzle (Andersson *et al.*, 2019 ▸).

Data were collected from LCP-grown microcrystals of C*c*O at BioMAX (Ursby *et al.*, 2020 ▸) using the 3D-printed flow cell. Data collection and structural refinement statistics are summarized in Table 1 ▸. Approximately 135 000 images were recorded using the 200 µm-diameter glass capillary and 263 000 images were recorded using the 100 µm-diameter glass capillary. The sample flow-rates were adjusted to achieve a similar velocity for the sample as it passed through the X-ray beam, and were 0.3 µl min^−1^ for 100 µm- and 1.2 µl min^−1^ for 200 µm-diameter glass capillaries to give a downwards velocity approximately equal to 0.64 mm s^−1^. The width of the X-ray beam was 20 µm, and this is much less than the diameter of either capillary. As such, the LCP volume sampled by the X-ray beam is only proportional to the path length of the X-ray beam through the capillary, and this was twice as long when using the 200 µm capillary as when using the 100 µm capillary. All things being equal, we expect that the indexing rate when using the 200 µm-diameter capillary should be approximately twice that of the 100 µm-diameter capillary. An indexing rate of 48.6% was observed for the larger capillary, whereas this was only 13.9% for the thinner capillary. It is possible that this difference (48.6/13.9 ≃ 3.5 ≠ 2) arises from more complex hydro­dynamics when using the thinner capillary with a viscous sample, since the sample tended to stop and start when operating at a low flow-rate of 0.3 µl min^−1^. However, we cannot rule out that this discrepancy may be the result of variations in the density of microcrystals between crystallization batches.

SSX data collected using the 100 µm-diameter flow cell could be processed to 2.72 Å, but the resolution limit was cut to 3.05 Å for this data set (Table 1 ▸) since the CC_1/2_ values were anomalous near 3.0 Å resolution, and therefore we did not analyse these data further. By contrast, data collected using the 200 µm-diameter flow cell were processed to 2.12 Å resolution, and appropriate *R*_work_ (16.1%) and *R*_free_ (18.8%) values were recovered (Table 1 ▸). Thus, despite the thicker sample support, a significantly higher resolution structure was recovered from approximately half the X-ray images when using 200 µm-diameter glass capillaries.

**Figure 3:**
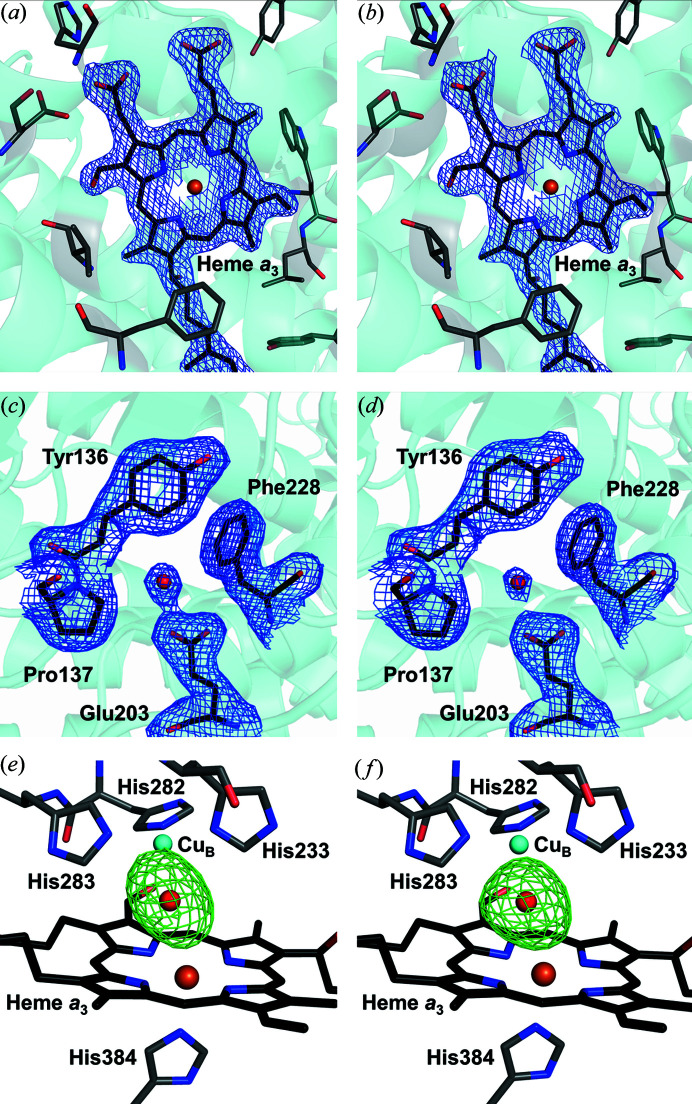
Comparison between the electron density recovered from SSX studies using a flow cell and SFX studies using a high-viscosity injector. (*a*) 2*F*_obs_ − *F*_calc_ electron density map (blue, contoured at 1.5σ) showing SSX electron density for the active site heme *a*_3_ of *ba*_3_-type C*c*O determined at 2.12 Å resolution recovered using the flow cell for sample injection. (*b*) 2*F*_obs_ − *F*_calc_ electron density map (blue, contoured at 1.5σ), showing SFX electron density for the active site heme *a*_3_ at 2.3 Å resolution recovered using a high-viscosity injector for sample injection (Andersson *et al.*, 2017 ▸). (*c*) 2*F*_obs_ − *F*_calc_ electron density map (blue, contoured at 1.0σ) showing SSX electron density for a representative glutamic acid residue within the protein. (*d*) 2*F*_obs_ − *F*_calc_ electron density map (blue, contoured at 1.0σ) showing SFX electron density for a representative glutamic acid residue within the protein. (*e*) *F*_obs_ − *F*_calc_ omit electron density map (green, contoured at 4.5σ), showing a slightly elongated electron density in the active site of the SSX structure. (*f*) *F*_obs_ − *F*_calc_ omit electron density map (green, contoured at 4.5σ) showing a slightly more spherical electron density in the active site of the SFX structure (Andersson *et al.*, 2017 ▸). For both omit maps the electron density map is calculated without modelling any ligand between the heme *a*_3_ iron and Cu_B_.

Comparison of the SSX structure obtained from 200 µm-diameter glass capillary using synchrotron radiation with an earlier structure (PDB entry 5ndc) obtained using XFEL radiation (Andersson *et al.*, 2017 ▸) (Table 1 ▸) shows similar-quality serial crystallography data with comparable resolution and similar values for *I*/σ, CC_1/2_, *R*_work_ and *R*_free_. Moreover, the 2*F*_obs_ − *F*_calc_ electron density maps appear to be very similar [Figs. 3 ▸(*a*)–3 ▸(*d*)]. Closer inspection of the *F*_obs_ − *F*_calc_ omit electron density maps between the heme *a*_3_ iron and the Cu_B_ copper atom of the binuclear centre, calculated without the addition of a ligand to either metal, also shows very similar electron density, although the SSX data yield a slightly more elliptical electron density feature in this position, whereas a more spherical electron density is observed for the SFX structure [Figs. 3 ▸(*e*) and 3 ▸(*f*)]. The peak height of the *F*_obs_ − *F*_calc_ omit electron density map was 1.15 e Å^−3^ for the SSX structure and 0.97 e Å^−3^ for the SFX structure. These values are much stronger than the average recovered for the ten strongest water molecules within the structure, which are 0.68 ± 0.04 e Å^−3^ in the SSX structure and 0.57 ± 0.05 e Å^−3^ in the SFX structure. We previously modelled either a water molecule or a hydroxide ion as a ligand to Cu_B_ (Andersson *et al.*, 2017 ▸), although a heavier ligand (Powers *et al.*, 1994 ▸) remains a distinct possibility. Another interpretation was given by the 1.8 Å resolution X-ray structure recovered from a single LCP-grown crystal of *ba*_3_-type C*c*O obtained by cryocrystallography (PDB entry 3s8g; Tiefenbrunn *et al.*, 2011 ▸), which revealed elongated *F*_obs_ − *F*_calc_ electron density at this position in the omit map with a peak value of 1.6 e Å^−3^, where a peroxide molecule was modelled in the active site. It was hypothesized that a peroxide ligand may be created due to X-ray induced radiation damage at low temperature. Density functional theory calculations on *ba*_3_-type C*c*O also suggest that an elongated density in the protein active site could arise from the overlap of electron densities of two closely spaced water molecules, each with partial occupancy (Han Du *et al.*, 2020 ▸).

### 3.3. Radiation damage considerations

Studies of X-ray induced radiation damage at room temperature have suggested that it is difficult to observe specific radiation damage from X-ray diffraction data sets obtained from a single crystal (Gotthard *et al.*, 2019 ▸) since, at room temperature, specific radiation damage occurs at a rate only marginally faster than global damage. SSX studies from a sequence of microcrystals have revealed both global and specific radiation damage (de la Mora *et al.*, 2020 ▸), with X-ray diffraction intensities observed to drop by half following a dose *D*_1/2_ of 570 kGy. In this work, each exposed microcrystal received an average room-temperature X-ray dose of 40 kGy, which is well below *D*_1/2_. Nevertheless, studies of X-ray induced damage at low temperature suggest that metals such as iron are sensitive to an X-ray dose as low as 40 kGy (Pfanzagl *et al.*, 2020 ▸), and C*c*O from *T. thermophilus* has been shown to be radiation sensitive (Liu *et al.*, 2009 ▸). Our structure does not show any signs of X-ray induced damage to radiation-sensitive residues such as glutamic acid residues [Fig. 3 ▸(*c*)]. However, we cannot rule out the possibility that the modest elongation of the *F*_obs_ − *F*_calc_ omit electron density feature observed between the active site heme *a*_3_ iron and the Cu_B_ copper atom [Fig. 3 ▸(*e*)] relative to that observed for the SFX structure [Fig. 3 ▸(*f*)] may be due to the partial photoreduction of these metal centres by X-rays. Experimental parameters such as the X-ray beam focus or degree of attenuation, as well as the sample flow-rate, should therefore be adjusted to ensure that the average X-ray dose per microcrystal does not exceed a specific threshold when there are scientific reasons to minimize the X-ray dose.

### 3.4. Background scattering from the borosilicate glass capillary

**Figure 4:**
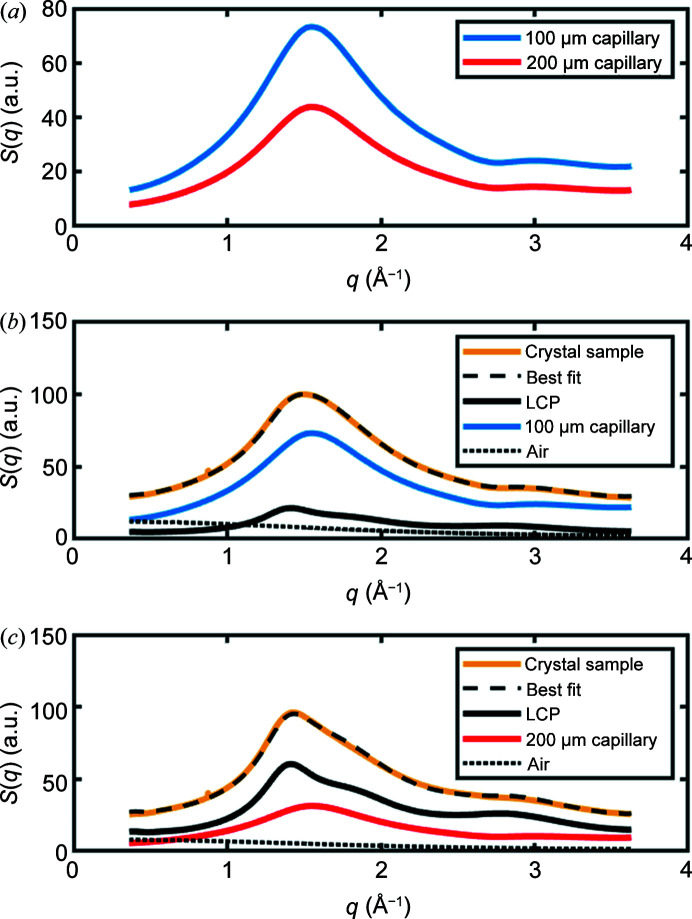
Analysis of the contributions to background X-ray scattering when using 100 and 200 µm-diameter borosilicate glass capillaries. (*a*) Measured X-ray scattering, *S*(*q*), from empty 100 µm (blue) and 200 µm (red) diameter borosilicate glass capillaries after azimuthal integration. The X-ray scattering has arbitrary units (a.u.) and 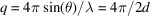 , where 2θ is the angle of deflection of the X-rays and 1/*d* is the resolution quoted in X-ray crystallography, such that *q* = 2.0 Å^−1^ corresponds approximately to 3.1 Å resolution. (*b*) Measured X-ray scattering from a 100 µm-diameter flow cell containing LCP-grown microcrystals of C*c*O (mustard) and the best decomposition (black dashed line) of this scattering into its three scattering components from LCP (black solid line), borosilicate glass (blue) and air (black dotted line). (*c*) Measured X-ray scattering from a 200 µm-diameter flow cell containing LCP-grown microcrystals of C*c*O (mustard) and the best decomposition (black dashed line) of this scattering into its three scattering components of LCP (black solid line), borosilicate glass (red) and air (black dotted line). Air measurements were made without any object at the sample position, whereas LCP scattering was recorded from a homogeneous LCP sample extruded below the glass of a 200 µm-diameter flow cell. Air scattering was removed from the LCP and glass in panels (*b*) and (*c*). A larger background contribution from the borosilicate glass is observed when using the 100 µm-diameter capillary relative to the 200 µm glass capillary.

Previous SSX studies using capillary-based flow cells reported an X-ray diffraction structure of lysozyme to 2.1 Å resolution using a 100 µm-diameter glass capillary for sample injection (Stellato *et al.*, 2014 ▸), and X-ray diffraction structures of lysozyme to 1.85 Å resolution and glucose isomerase to 1.70 Å resolution using a 400 µm-diameter quartz capillary for sample injection (Nam, 2020 ▸). In our SSX studies of microcrystals of C*c*O, we used borosilicate glass capillaries of 100 and 200 µm in diameter. Prior to data collection, we anticipated that the thinner capillary would yield higher-quality X-ray diffraction data due to lower background X-ray scattering, yet considerably better quality X-ray diffraction data emerged when using the 200 µm capillary (Table 1 ▸). To understand what may underpin this observation, we examined the X-ray scattering contributions from both 100 and 200 µm-diameter capillaries when empty [Fig. 4 ▸(*a*)]. Although both capillaries had a nominal borosilicate glass wall thickness of 10 µm, it was apparent that the X-ray scattering from the glass when using the 100 µm-diameter capillary was approximately twice that of the 200 µm capillary. This may be due to the process of pulling the melted glass leading to thicker glass walls for thinner (and therefore more fragile) capillaries. We further decomposed the X-ray scattering from both 100 and 200 µm-diameter capillaries into the scattering from their respective components by comparing with appropriate reference measurements [Figs. 4 ▸(*b*) and 4 ▸(*c*)]. It is striking that the X-ray scattering from the LCP is much reduced when using a 100 µm capillary relative to a 200 µm capillary, yet the stronger X-ray scattering from the glass walls associated with the thinner capillary cancels out this benefit. We therefore recommend using the 200 µm capillary as an initial choice, but acknowledge that there may be cases where the 100 µm capillary is advantageous, like in studies of well diffracting lysozyme microcrystals (Stellato *et al.*, 2014 ▸) or when it is difficult to obtain large quantities of sample and therefore a slower flow-rate is advantageous.

### 3.5. Using the 3D-printed flow cell for in-line spectroscopy

**Figure 5:**
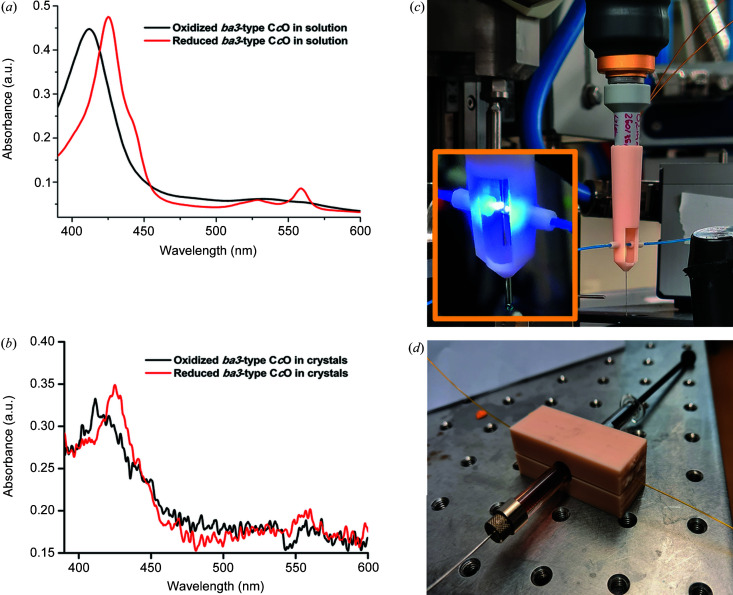
Spectroscopic measurements using the flow cell. (*a*) Absorbance spectra recorded from samples of purified *ba*_3_-type C*c*O from *T. thermophilus* in solution. Red: C*c*O is purged of oxygen and reduced. Black: C*c*O in the oxidized state. (*b*) Absorbance spectra recorded from a slurry of C*c*O microcrystals held in a flow cell. Red: C*c*O is purged of oxygen and reduced. Black: C*c*O in the oxidized state. (*c*) Modified catcher, used to hold aligned optical fibres incoming from either side of the X-ray transparent capillary. This either allows absorption spectra to be recorded from samples as they pass by or allows light to be transported to the sample to activate photo-sensitive samples (inset). (*d*) Device for measuring absorption spectra from samples suspended in a glass syringe prior to injection using the flow cell. This is achieved using aligned optical fibres incoming from either side of the transparent syringe.

Another potential application for the 3D-printed flow cell is the UV–Vis spectroscopic characterization of proteins in both solution and crystalline form, since this allows experimental protocols to be evaluated within the same sample environment in advance of synchrotron-based time-resolved SSX studies. Fig. 5 ▸ shows absorption spectra recovered from the oxidized and reduced forms of *ba*_3_-type C*c*O in solution [Fig. 5 ▸(*a*)] and in crystalline form [Fig. 5 ▸(*b*)]. Monitoring of the absorption spectra from continuously flowing protein in solution revealed that an oxygen-free environment could be maintained in the whole system for several hours, from the syringe and into the capillary flow cell. As illustrated in Fig. 5 ▸(*c*), the catcher can be adapted to hold optical fibres which are suitable either for in-line spectroscopic measurements on samples flowing through the capillary or for transporting light to illuminate samples that may be photo-activated. Moreover, a conceptually related device can be used to obtain spectra directly from a crystal-loaded glass syringe [Fig. 5 ▸(*d*)]. These tools thus create opportunities to perform SSX experiments while optically monitoring the state of microcrystals both in the syringe and in the flowing capillary.

## 4. Conclusions

Since the first demonstration experiments in 2014 (Gati *et al.*, 2014 ▸), synchrotron-based serial crystallography has held the potential to expand the serial crystallography user community owing to the much larger number of macromolecular crystallography beamlines supporting user experiments at synchrotrons than at XFELs. Entry barriers for inexperienced users, however, remain high. A variety of fixed-target (Sherrell *et al.*, 2015 ▸; Mueller *et al.*, 2015 ▸; Mehrabi *et al.*, 2019*a* ▸, 2020 ▸; Schulz *et al.*, 2018 ▸; Suga *et al.*, 2019 ▸), flowing-microjet or microfluidic technologies (Weierstall *et al.*, 2014 ▸, 2012 ▸; DePonte *et al.*, 2008 ▸; Vakili *et al.*, 2022 ▸; Weinert *et al.*, 2017 ▸), and tape conveyor belts (Fuller *et al.*, 2017 ▸; Beyerlein *et al.*, 2017 ▸) are in use and reflect developments and adaptions of instrumentation over the years. Flow cell systems offer a simple alternative when using synchrotron radiation, and are reminiscent of a previous age of single-crystal room-temperature crystallography (Basavappa *et al.*, 2003 ▸). Existing designs, however, require a separate sample stage for alignment and manipulation (Stellato *et al.*, 2014 ▸; Nam, 2020 ▸).

In this work we have developed a lightweight, magnetically mounted flow cell design which takes advantage of the tools for sample alignment that are integrated into every macromolecular crystallography beamline worldwide, while respecting the magnetic-mount standards developed for cryocrystallography. By designing the system to be compatible with existing standards, the flow cell can be rapidly installed and aligned without perturbing other features of a standard macromolecular crystallography beamline. Although the data presented here were collected using a vertical mount from above at BioMAX [Fig. 2 ▸(*a*)], the system may be modified for vertical mounting from below [Fig. 2 ▸(*d*)] or using a horizontal mount [Fig. 2 ▸(*e*)]. The system can be used in-house for testing the flow of microcrystals in advance of travel to a synchrotron radiation user facility, or for spectroscopic characterization of samples in advance or during an experiment (Fig. 5 ▸), including when working with oxygen-sensitive samples. Since glass capillaries are transparent to light, the flow cell is suitable for time-resolved diffraction studies of light-sensitive proteins (Branden & Neutze, 2021 ▸; Tenboer *et al.*, 2014 ▸). By incorporating multiple fused silica capillary tubing inputs into the device and extending the design to incorporate mixing channels within the 3D-printed base, enzymatic reactions may also be initiated by mixing within the flow cell, as has been successfully demonstrated with several other mix-and-inject devices (Beyerlein *et al.*, 2017 ▸; Calvey *et al.*, 2019 ▸, 2016 ▸; Dasgupta *et al.*, 2019 ▸; Monteiro *et al.*, 2020 ▸; Olmos *et al.*, 2018 ▸; Stagno *et al.*, 2017 ▸; Wang *et al.*, 2014 ▸). Overall, these designs aim to lower entry barriers for non-expert users and provide tools that may allow any macromolecular crystallography beamline with a rapid-readout X-ray detector to offer SSX to their users.

The atomic coordinates and structure factor files for data collected using a 200 µm-diameter capillary have been deposited in the Protein Data Bank (https://www.pdb.org) under PDB entry 8hua.

## Supplementary Material

PDB reference: *ba*3-type cytochrome *c* oxidase from *Thermus thermophilus*, 8hua

## Figures and Tables

**Table 1:** Data collection and refinement statistics

	100 µm glass capillary	200 µm glass capillary	SFX structure
Data collection			
PDB entry		8hua	5ndc
Temperature (K)	293	293	293
Space group	*C*121	*C*121	*C*121
Cell dimensions			
*a*, *b*, *c* (Å)	145.4, 100.2, 96.6	146.1, 100.2, 96.6	145.9, 100.3, 96.6
α, β, γ (°)	90, 126.8, 90	90, 126.8, 90	90, 126.8, 90
Resolution (Å)†	25.2–3.05 (3.16–3.05)	25.4–2.12 (2.20–2.12)	36.4–2.30 (2.34–2.30)
*R*_split_ (%)† ‡	9.1 (65.9)	10.4 (78.1)	19.4 (120)
〈*I*/σ*I*〉†	8.2 (1.5)	7.0 (1.2)	3.7 (1.02)
CC_1/2_ †	99.1 (69.2)	99.1 (55.9)	95.6 (36.6)
Completeness (%)	100 (100)	100 (100)	100 (100)
Multiplicity†	115.8 (76.8)	124.8 (82.7)	36.8 (14.7)
No. of collected images	263118	135000	87057
No. of indexed patterns	36702	65638	8211
Indexing rate (%) (indexed patterns/collected images)	13.9	48.6	9.4
Total No. of reflections	3539032	7887328	1864107
No. of unique reflections	30553	63185	50602

Refinement			
Resolution		25.8–2.12	36.4–2.3
*R*_work_/*R*_free_ (%)		16.1/18.8	16.2/19.8
No. of atoms		12953	6386
Average *B* factor (Å^2^)		52	43.9
R.m.s deviations			
Bond lengths (Å)		0.013	0.012
Bond angles (°)		1.84	1.61

† Values in parentheses correspond to those of the highest-resolution shell.

‡