# A simple goniometer-compatible flow cell for serial synchrotron X-ray crystallography. Corrigendum

**DOI:** 10.1107/S1600576725009215

**Published:** 2025-10-24

**Authors:** Swagatha Ghosh, Doris Zorić, Peter Dahl, Monika Bjelčić, Jonatan Johannesson, Emil Sandelin, Per Borjesson, Alexander Björling, Analia Banacore, Petra Edlund, Oskar Aurelius, Mirko Milas, Jie Nan, Anastasya Shilova, Ana Gonzalez, Uwe Mueller, Gisela Brändén, Richard Neutze

**Affiliations:** a Department of Chemistry and Molecular Biology University of Gothenburg Medicinaregatan 9C 40530Gothenburg Sweden; b MAX IV Laboratory Lund University Fotongatan 2 224 84Lund Sweden; c Diamond Light Source Harwell Science and Innovation Campus DidcotOX11 0DE United Kingdom; d Macromolecular Crystallography Group Helmholtz-Zentrum Berlin Albert-Einstein-Strasse 15 12489Berlin Germany

**Keywords:** serial synchrotron X-raycrystallography, macromolecular crystallography, cytochrome *c* oxidase, goniometer-compatible flow cells

## Abstract

Information concerning sample preparation in the article by Ghosh *et al.* [*J. Appl. Cryst.* (2023), **56**, 449–460] is clarified.

In the article by Ghosh *et al.* (2023 ▸), the microcrystal preparations used when measuring the absorption spectra shown in Fig. 5(*b*) were not accurately described. Microcrystalline samples were reduced by the addition of dithiothreitol and mixed with a photocage (Sandelin *et al.*, 2024 ▸), and the absorption spectrum for the reduced state was recorded. The sample was translated, a laser flash of 355 nm was used to release dioxygen, and an absorption spectrum for the oxidized state was recorded after a delay of 1 ms. The absorption contribution of the photocage was subtracted from both spectra. Spectra from detergent-solubilized samples of cytochrome *c* oxidase (C*c*O) [Fig. 5(*a*)] were prepared as described: the oxidized spectrum is recorded from C*c*O samples as isolated, the reduced spectrum is recorded after treatment with sodium di­thio­nite under anaerobic conditions, and no photocage was used in those measurements.
